# Optimization of Cryogenic Gas Separation Systems Based on Exergetic Analysis—The Claude–Heylandt Cycle for Oxygen Separation

**DOI:** 10.3390/e28010125

**Published:** 2026-01-21

**Authors:** Dănuț-Cristian Urduza, Lavinia Grosu, Alexandru Serban, Adalia Andreea Percembli (Chelmuș), Alexandru Dobrovicescu

**Affiliations:** 1 Department of Engineering Thermodynamics, National University of Science and Technology Politehnica București, 060042 Bucharest, Romania; danut.urduza@stud.mec.upb.ro (D.-C.U.); alexandru.serban@criomecsa.ro (A.S.); 2 Laboratoire Energétique Mécanique Electromagnétisme (LEME), IUT de Ville d’Avray, 92410 Ville d’Avray, France; mgrosu@parisnanterre.fr

**Keywords:** Claude–Heylandt cycle, air liquefaction, exergy analysis, oxygen separation

## Abstract

In cryogenic air liquefaction systems, a major share of the mechanical energy consumption is associated with exergy destruction caused by heat transfer in recuperative heat exchangers. This study investigated the exergetic optimization of cryogenic gas separation systems by focusing on the Claude–Heylandt cycle as an advanced structural modification of the classical Linde–Hampson scheme. An exergy-based analysis demonstrates that minimizing mechanical energy consumption requires a progressive reduction in the temperature difference between the hot forward stream and the cold returning stream toward the cold end of the heat exchanger. This condition was achieved by extracting a fraction of the high-pressure stream and expanding it in a parallel expander, thereby creating a controlled imbalance in the heat capacities between the two streams. The proposed configuration reduces the share of exergy destruction associated with heat transfer in the recuperative heat exchanger from 14% to 3.5%, leading to a fourfold increase in the exergetic efficiency, together with a 3.6-fold increase in the liquefied air fraction compared with the Linde–Hampson cycle operating under identical conditions. The effects of key decision parameters, including the compression pressure, imposed temperature differences, and expander inlet temperature, were systematically analyzed. The study was further extended by integrating an air separation column into the Claude–Heylandt cycle and optimizing its configuration based on entropy generation minimization. The optimal liquid-air feeding height and threshold number of rectification trays were identified, beyond which further structural complexity yielded no thermodynamic benefit. The results highlight the effectiveness of exergy-based optimization as a unified design criterion for both cryogenic liquefaction and separation processes.

## 1. Introduction

The continuous increase in global energy demand has intensified concerns regarding improving the efficiency of industrial processes. In the context of European objectives for transitioning toward low-carbon systems [1,2] promoted through initiatives such as the European Green Deal [3], the optimization of processes characterized by high energy consumption has become a priority. Within this framework, the reduction in energy consumption and enhancement of the efficiency of cryogenic gas-liquefaction installations represent major directions of scientific interest [4,5,6]. The present study continues with a series of investigations dedicated to the analysis and optimization of the fundamental thermodynamic processes that govern these installations.

In the previous stage, a detailed comparative analysis was performed on the Linde–Hampson cycles [7], through which both the quantitative and qualitative aspects of the internal thermodynamic processes were evaluated. This analysis enabled the identification of the main sources of irreversibility, quantification of exergy destruction, and formulation of structural optimization solutions. Continuing this approach, the present study focuses on the Claude–Heylandt cycle. The evolution toward the Claude cycle introduced a significant improvement compared to the Linde–Hampson cycle by integrating an expander in parallel with the throttle valve. This configuration optimizes the distribution of gas flow rates, reduces exergy destruction, and enhances the overall performance of the system. Simultaneously, the approach includes fractioning of the forward flow rate, a technique that redirects a part of the gas flow toward the expander, thereby allowing an additional cooling contribution in the returning stream. Several studies [8,9] in the specialized literature confirm the relevance and continuity of this optimization direction, including the work of David Berstad et al. [10], in which the authors performed an exergy analysis of an industrial-scale Claude hydrogen-liquefaction system with precooling based on a mixed-refrigerant cycle. Their study provides an overview of the distribution of irreversibility within each component of the process. It shows that more than 90% of the total irreversibility of the process originates from the Claude cycle itself, the largest exergy destructions being associated with the compression and intermediate cooling of hydrogen (39%), the cryogenic heat exchangers (21%), and the expansion turbines (13%). The interest in the Claude cycle is also shown in the study conducted by Tamarona et al. [11], which, in addition to thermodynamic analysis, also performs an economic evaluation to estimate the specific liquefaction costs and highlight the main aspects that influence the functionality of the process.

Khodaparast et al. [12] proposed two configurations of hydrogen-liquefaction installations, based on the Claude and reversed Brayton cycles, integrated with an organic Rankine cycle supplied with geothermal energy to reduce the energy consumption and precooling the hydrogen at the inlet of the installation. The results showed that the system based on the Claude cycle provides superior performance compared with the one using the reversed Brayton cycle, achieving a specific consumption of 14.8 kWh/kg and an overall exergetic efficiency of 71%, compared with 17.2 kWh/kg and 62% in the alternative case. The results highlight the superiority of the Claude cycle, which enables a more efficient supply of liquid nitrogen with minimal energy losses.

An increased interest in the optimization of the Claude cycle was also highlighted in the study by Lee et al. [13], in which the authors integrated into a single system the processes of hydrogen production through steam methane reforming, hydrogen liquefaction, and CO_2_ liquefaction, using a modified Claude cycle that reduced the specific energy consumption to 6.15 kWh/kg LH_2_, approximately 47% lower than in the reference process.

In their study, Cardella et al. [14] proposed a high-pressure Claude hydrogen cycle assisted by a precooling mixed-refrigerant cycle (MRC), demonstrating the possibility of reducing energy consumption and improving exergetic efficiency.

In the installation proposed by Yan et al. [15], the Claude cycle was coupled with a precooling mixed-refrigerant cycle (MRC), steam methane reforming (SMR) for hydrogen generation, a CO_2_ refrigeration cycle and a solar absorption system (ARS), all of which contributed to the enhancement of the energetic performance. The authors highlighted that, in this configuration, there was a reduction of the specific energy consumption to 5.22 kWh/kg and an exergetic efficiency of 62.2%.

Thomas et al. [16] performed an exergy analysis of a helium-liquefaction system based on the modified Claude cycle, highlighting that increasing the flow rate through the first expander amplifies the exergy destruction at high temperatures, whereas the addition of supplementary expansion stages can reduce this destruction, thereby improving the overall performance of the cycle.

Kanoglu et al. [17] proposed a system in which the Claude cycle was integrated with a precooling unit supplied with geothermal energy.

All these studies demonstrate the continuity and relevance of the optimization direction of the Claude cycle in an effort to reduce the exergy destruction and increase the overall performance.

Optimization of cryogenic gas-separation systems represents a field of interest in current research, particularly with regard to the separation of different gases such as oxygen. The production of liquid air and the extraction of oxygen are of special importance in the industry, which is why numerous authors have directed their studies toward this area [18,19,20], including Hamayun et al. [21], who emphasized the importance of oxygen and its widespread use in the chemical industry, steel production, semiconductor manufacturing, and the health sector in their study.

Bucșa et al. [22] propose an analysis based on the exergetic evaluation of a cryogenic air-separation installation with a production capacity of high-purity gaseous oxygen (above 98.5%). The study provides a detailed model for assessing the process by correlating the decisional and structural parameters of the installation, defining for each functional zone the exergetic product as well as the main sources of exergy destruction. Thus, the authors identified the compression zone as having the highest share of exergy destruction (37%). However, a significant contribution to the total exergy destruction was also observed in the high-pressure rectification column, where the second-largest share of 8.3% was recorded, highlighting the importance of this stage in the overall efficiency of the process. These results are consistent with observations from previous studies [23,24,25]. Ebrahimi et al. [23] emphasized the high share of destruction in compression and rectification columns, underlining the need for the simultaneous optimization of the heat exchanger network (including through Pinch Analysis) and the distillation section.

This direction is supported by recent studies by Shingan et al. [26] and Liu et al. [27], who showed that the air-compression stage represents the main source of exergy destruction in cryogenic air-separation installations. Both works are focused on process optimization through energy-integration methods: Shingan et al. [26] proposed the recovery of waste heat by coupling the installation with LNG regasification processes, achieving a reduction of more than 58% in energy consumption, while Liu et al. [27] developed a scheme with liquid-nitrogen storage and the recovery of compression heat, which led to a 36.4% decrease in the levelized cost of energy compared with conventional processes. These perspectives are complemented by the study conducted by Ioniță et al. [28], who analyze an air-separation installation, determining an overall exergetic efficiency of 14% and highlighting that the share of exergy losses associated with the heat discharged through the compressor cooling system represents approximately 21% of the electrical consumption of the installation. The authors examined the possibility of recovering waste heat using an ORC cycle proposed for converting it into mechanical work by comparing several organic refrigerants (R-245fa, R123, n-pentane, and R717). The results showed that R717, due to the possibility of vapor superheating, provides the best efficiency, equivalent to a saving of approximately 6% of the electrical energy of the installation. Continuing the research direction, Mtogo et al. [29,30] performed, through Aspen Plus simulations, an exergy analysis of different rectification-column configurations for azeotropic mixtures, highlighting the way in which the operating conditions and the nature of the various azeotropic agents influence the distribution of exergy destruction throughout the system.

In addition, Huo et al. [31] focused on the optimization of a cryogenic air-separation installation (ASU) by analyzing the use of a cryogenic expander instead of the throttle valve. Following this study, it was found that the introduction of the expander reduced the energy consumption for producing one unit of oxygen by approximately 2.12% and led to an increase in the total exergetic efficiency by approximately 1%.

Although extensive research has been devoted to the exergetic analysis and optimization of cryogenic liquefaction and air separation systems, most existing studies have addressed individual subsystems, such as compression, heat exchange, or rectification, in an isolated manner. Limited attention has been paid to the explicit relationship between the temperature difference profile in recuperative heat exchangers, structural configuration of the liquefaction cycle, and consistent integration of these aspects with the exergetic design of the separation column.

Therefore, the present paper proposes a strategy for analysis and optimization. In the first stage, the investigation focused on the exergetic analysis of the Claude–Heylandt cycle. In the study, the reverse Pinch analysis was applied, gradually reducing the imposed temperature difference and recalculating the utilities until they vanished, the point at which the external utilities vanished, indicating the real minimum temperature difference between the two streams in the recuperative heat exchanger. The analysis highlighted the potential of the controlled imbalance of the heat capacities between the hot and cold streams in the recuperative heat exchangers—by extracting a gas flow rate from the hot high-pressure stream and expanding it in an expander placed in parallel with the throttle valve.

Following this optimization step, the second stage extends the configuration by coupling the Claude–Heylandt cycle with an air-separation column, introducing exergetic design criteria based on minimizing the entropy generated for two essential constructive decisions: (i) the optimal height of the liquid-air feeding point of the rectification column and (ii) the number of rectification trays. It was shown that there exists a threshold number of trays beyond which increasing their number becomes thermodynamically insignificant, and that raising the height of the feeding level increases the efficiency of the system.

The novelty of the present work lies in the unified application of exergy analysis to both the Claude–Heylandt liquefaction cycle and the air separation column, with optimization understood in the thermodynamic sense as the identification of system architectures and operating conditions that minimize entropy generation and exergy destruction. By explicitly linking the Claude–Heylandt architecture to the optimal temperature-difference profile in recuperative heat exchangers and extending the analysis to the rectification column based on entropy generation criteria, this study provides a coherent strategy for the integrated optimization of cryogenic liquefaction–separation systems.

**Figure 1:**
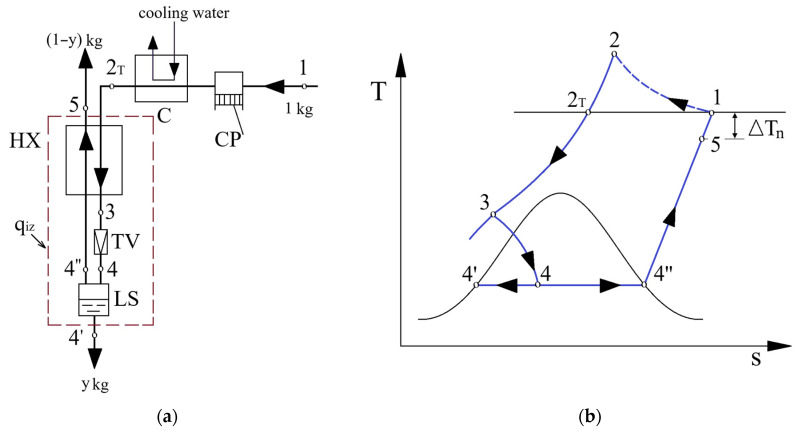
The basic Linde–Hampson liquefaction cycle: (**a**) process flow diagram; (**b**) cycle thermodynamic representation in the T–s diagram.

The exergetic analysis of the one-stage Linde–Hampson liquefaction cycle [7] (Figure 1) highlighted that the share of exergy destruction in the global mechanical-energy consumption of the installation, associated with the irreversibility of the heat-transfer process is (Table 1)

(1) $$\psi_{\Delta T}=100\frac{I_{\Delta T}}{\left|w_{cp}\right|}=14\%,$$

where $I_{\Delta T}$ represents the exergy destruction and, $\left|w_{cp}\right|$ is the compressor mechanical work consumed for 1 kg of air.

To identify solutions for reducing exergy consumption in the recuperative heat exchanger, the mechanism of extracting heat from the high-pressure forward stream and transferring it to the ambient environment by the cold returning stream was examined.

## 2. The Optimal Temperature Difference in the Heat Exchanger

From the analysis of the operation of the heat exchanger of the Linde-type liquefaction installation with one throttling, it is observed that, when the throttle valve is eliminated, the difference between the temperature of the gas in the forward stream at the outlet of the heat exchanger and the temperature of the cold region implies continuous penetration into the cold zone of the heat flux transferred through the forward stream.

Maintaining the temperature of the cold region constant requires the discharge of this amount of heat into the ambient environment, which leads to a minimum mechanical-work consumption equal to the exergy of the heat transferred at the temperature of the cold zone.

**Figure 2:**
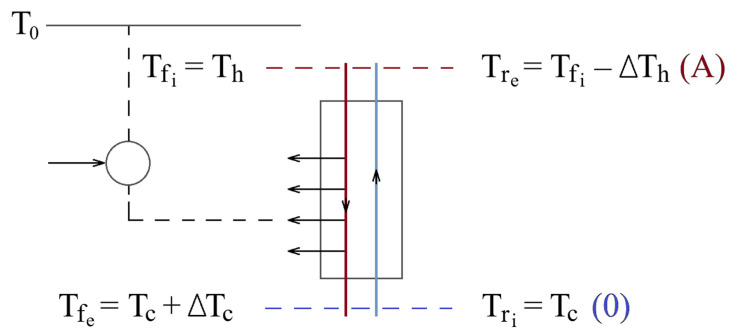
The precooling of the gas in the forward stream by means of ideal refrigeration machines operating between an intermediate temperature T and the ambient temperature T_0_.

A reduction in the minimum $w_{cp}$ required to extract the heat transported by the high-pressure stream into the cold region of the installation could be achieved by partially discharging this heat, along the path of the forward stream, before it reaches the cold zone, by means of refrigeration machines operating on a reversed Carnot cycle (Figure 2) [32].

It should be noted that the difference between the temperature of the gas in the forward stream at the outlet of the recuperative heat exchanger $\left(T_{f,e}\right)$ and the temperature of the cold region $\left(T_{c}\right)$ represents the temperature difference between the forward and returning streams at the cold end of the heat exchanger $\left(\Delta T_{c}=T_{f,e}-T_{r,i}\right)$ (Figure 2).

The described process, namely, the penetration of heat through the gas in the forward stream from the hot end of the heat-exchange apparatus toward the cold end, is characteristic not only of the recuperative exchanger of the throttling stage but also of any recuperative exchanger of the cryogenic installation that occurs in every section of the heat-transfer surface.

Under the conditions in which an attempt is made to reduce the amount of heat carried by the forward stream toward the colder sections of the heat exchanger, through the intermediate precooling of the gas stream by means of ideal refrigeration machines (Figure 2), the minimum mechanical work consumed for discharging the intermediate amounts of heat and the heat that is transferred at the cold end of the exchanger represents the sum of the exergies of these heat quantities, whose values are determined by the ambient temperature T_0_ and by the temperatures T at which the heat-rejection process took place.

Denoting by ${\dot{Q}}_{c}$ the absolute value of the heat flux transferred to the cold region, and by $\delta \dot{Q}$ the absolute, elementary values of the heat fluxes extracted from the forward stream through precooling at a temperature level T situated between $T_{c}$ and $T_{h}$ (the temperatures at the cold and hot ends of the apparatus, respectively), the calculation relation of the minimum power consumed to reject to the ambient the heat carried by the forward stream toward the cold end of the recuperative heat exchanger is

(2) $$P_{min}=\left|{\dot{Ex}}_{Q_{c}}\right|+\sum \left|{\dot{Ex}}_{Q_{i}}\right|={\dot{Q}}_{c}\left(\frac{T_{0}}{T_{c}}-1\right)+\int_{T_{c}}^{T_{h}}\delta \dot{Q}\left(\frac{T_{0}}{T}-1\right).$$

The integral representing the sum of the exergies of the intermediate heat quantities extracted from the forward stream can be expressed as follows:

(3) $$\int_{T_{c}}^{T_{h}}\left(\frac{T_{0}}{T}-1\right)\delta \dot{Q}=\int_{T_{c}}^{T_{h}}\left(\frac{T_{0}}{T}-1\right)\frac{\delta \dot{Q}}{dT}dT.$$

Noting that

(4) $$\left(\frac{T_{0}}{T}-1\right)=f\left(T\right),\;and$$

(5) $$\dot{Q}\left(T\right)=g\left(T\right),$$

integral (3) can be rewritten as

(6) $$\int_{T_{c}}^{T_{h}}\left(\frac{T_{0}}{T}-1\right)\frac{\delta \dot{Q}}{dT}dT=\int_{T_{c}}^{T_{h}}f(T)\cdot g^{\prime }(T)dT,$$

which can be integrated by parts.

By integrating by parts, we obtain the expression for the minimum power consumed for the intermediate precooling in the forward gas stream as follows:

(7) $$\int_{T_{c}}^{T_{h}}\left(\frac{T_{0}}{T}-1\right)\frac{\delta \dot{Q}}{dT}dT={\dot{Q}\left(\frac{T_{0}}{T}-1\right)|}_{T_{c}}^{T_{h}}+\int_{T_{c}}^{T_{h}}\dot{Q}\frac{T_{0}}{T^{2}}dT={\dot{Q}}_{h}\left(\frac{T_{0}}{T_{h}}-1\right)-{\dot{Q}}_{c}\left(\frac{T_{0}}{T_{c}}-1\right)+T_{0}\int_{T_{c}}^{T_{h}}\frac{\dot{Q}}{T^{2}}dT,$$

By substituting integral (7) into relation (2), one obtains

(8) $$P_{min}={\dot{Q}}_{h}\left(\frac{T_{0}}{T_{h}}-1\right)+T_{0}\int_{T_{c}}^{T_{h}}\frac{\dot{Q}}{T^{2}}dT.$$

Obviously, if the hot end of the HX is at the ambient temperature $\left(T_{h}=T_{0}\right)$ (the case of single throttling Linde cycle—Figure 1), Equation (8) can be written as

(9) $$P_{min}=T_{0}\int_{T_{c}}^{T_{h}}\frac{\dot{Q}}{T^{2}}dT.$$

The minimization of the (minimum) $w_{cp}$ required to reject the heat carried by the forward stream implies the determination of the optimal heat flux $\dot{Q}(T)$ that minimizes the value of the integral expression (9)

(10) $$J=\int_{T_{c}}^{T_{h}}\frac{\dot{Q}}{T^{2}}dT$$

It is observed that

(11) $$-\frac{\dot{Q}}{T^{2}}dT=\delta {\dot{S}}_{gen},$$

because of the heat transfer at a finite temperature difference between the hot forward and cold return streams.

The minimization of the integral J (Equation (10)) reduces to the minimization of a function of two variables

(12) $$f\left(\dot{Q},T\right)=\frac{\dot{Q}}{T^{2}}.$$

The necessary condition for an extremum is the existence of the stationary point of this two-variable function

(13) $$df=\frac{\partial f}{\partial \dot{Q}}\delta \dot{Q}+\frac{\partial f}{\partial T}dT=0.$$

If $\dot{Q}$ and T are independent, then the necessary condition for stationarity is

(14) $$\frac{\partial f}{\partial \dot{Q}}=0\;and\;\frac{\partial f}{\partial T}=0,$$

which satisfies Equation (13) for any $\dot{Q}$ and T (independent). However, $\dot{Q}(T)$ and T are not independent. A dependence relation between them must be found so that one can be substituted as a function of the other, and Equation (13) becomes an equation with a single variable.

It is observed that the total cost of the installation, consisting of the invested capital and operating costs, generally forms the objective function to be minimized. Consequently, the heat-transfer surface can be considered as specified, which constitutes the dependence relationship between $\dot{Q}(T)$ and T

(15) $$A=\int_{T_{c}}^{T_{h}}\frac{{\left({\dot{m}}_{f}\cdot c_{p,f}\right)}^{2}}{U\cdot \dot{Q}}dT.$$

With this observation, the minimization of the objective function (10) is attempted under constraint (15), by applying the Lagrange multipliers method.

The problem reduces to the minimization of the Lagrangian

(16) $$L\left(\dot{Q},T\right)=f\left(\dot{Q},T\right)+\lambda \cdot g\left(\dot{Q},T\right),$$

where λ is the Lagrange multiplier, whose value, determined from the extremum condition of the function L (Equation (16)), transforms the optimization procedure of the function $f\left(\dot{Q},T\right)$ with the constraint of specifying the surface A into an unconstrained one, and the function $g\left(\dot{Q},T\right)$ is

(17) $$g\left(\dot{Q},T\right)=\frac{{\left({\dot{m}}_{f}\cdot c_{p,f}\right)}^{2}}{U\cdot \dot{Q}}-A.$$

The necessary condition for optimality is

(18) $$\frac{\partial L}{\partial \dot{Q}}\delta \dot{Q}+\frac{\partial L}{\partial T}dT=0,$$

for which the stationary-point condition is

(19) $$\{\begin{array}{c} \frac{\partial L}{\partial \dot{Q}}=0\\ \frac{\partial L}{\partial T}=0\\ A=\int_{T_{c}}^{T_{h}}\frac{{\left({\dot{m}}_{f}\cdot c_{p,f}\right)}^{2}}{U\cdot \dot{Q}}dT \end{array}.$$

The stationary-point conditions imposed by system (19), are

(20) $$\frac{\partial L}{\partial \dot{Q}}=\frac{1}{T^{2}}-\lambda \frac{{\left({\dot{m}}_{f}\cdot c_{p,f}\right)}^{2}}{U\cdot {\dot{Q}}^{2}}=0,$$

(21) $$\frac{\partial L}{\partial T}=-\frac{2\dot{Q}}{T}=0.$$

Equation (21) introduces trivial conditions T→∞ or $\dot{Q}=0$, which are of no interest.

From Equation (20), one obtains

(22) $$\frac{1}{T^{2}}=\lambda \frac{{\left({\dot{m}}_{f}\cdot c_{p,f}\right)}^{2}}{U\cdot {\dot{Q}}^{2}},$$

and finally

(23) $${\dot{Q}}_{opt}=\lambda^{1/2}\frac{{\dot{m}}_{f}\cdot c_{p,f}\cdot T}{U^{1/2}}.$$

For the calculation of λ, which makes the constrained optimization an unconstrained one, the optimal flux ${\dot{Q}}_{opt}$, which extremizes the function $f(\dot{Q},T)$, must satisfy Equation (15)

(24) $$\lambda^{1/2}=\frac{1}{A}\int_{T_{c}}^{T_{h}}\frac{{\dot{m}}_{f}\cdot c_{p,f}}{U^{1/2}\cdot T}dT.$$

After integration, it follows that

(25) $$\lambda^{1/2}=\frac{1}{A}\frac{{\dot{m}}_{f}\cdot c_{p,f}}{U^{1/2}}ln\frac{T_{h}}{T_{c}}.$$

By substituting the value of λ (Equation (24)) into the expression for the optimal heat flux, we obtain

(26) $${\dot{Q}}_{opt}=T\frac{{\left({\dot{m}}_{f}\cdot c_{p,f}\right)}^{2}ln\frac{T_{h}}{T_{c}}}{A\cdot U}.$$

From the analysis of Equation (26), it is noted that the minimization of the power consumption required to reject the heat is achieved under the conditions in which the heat flux carried by the forward stream toward the cold end of the recuperative heat exchanger practically decreases with temperature and with a decrease in the gas flow rate in the forward stream.

Noting that

(27) $${\dot{Q}}_{opt}=U\cdot A\cdot {\Delta T}_{opt},$$

it follows that ${\dot{Q}}_{opt}$ is proportional to the optimal temperature difference between the two streams, ${\Delta T}_{opt}$, which must decrease toward the cold end of the recuperative heat exchanger.

A reduction in the temperature difference ΔT between the hot forward stream and the cold returning stream toward the cold end of the recuperative heat exchanger can be achieved either by precooling the forward stream or by reducing the gas flow rate in the forward stream.

An alternative approach would consist of increasing the amount of refrigeration in the returning stream, so that it absorbs a larger quantity of heat from the forward stream, simultaneously with the reduction in the flow rate in the forward stream.

The additional refrigeration required in the return stream can be achieved by introducing a cold stream obtained through the expansion of the gas in the expander.

If the gas expanded in the expander is a fraction of the gas extracted from the forward stream, the second objective is also achieved: reduction in the flow rate in the forward stream.

## 3. The Cryogenic Liquefaction Claude–Heylandt Cycle

The injection into the returning stream of a cold gas stream obtained through expansion in an expander provides an additional refrigeration contribution to the recuperative heat exchanger.

The solution for reducing the gas flow rate in the forward stream as the processes proceed toward lower temperatures is consistent with the optimal profile of the temperature difference between the forward and returning streams in the recuperative heat exchangers.

All these considerations converge toward an optimal solution, indicating the need for structural changes to the system architecture by introducing an expansion of the hot gas in an expander in parallel.

### 3.1. System Description

**Figure 3:**
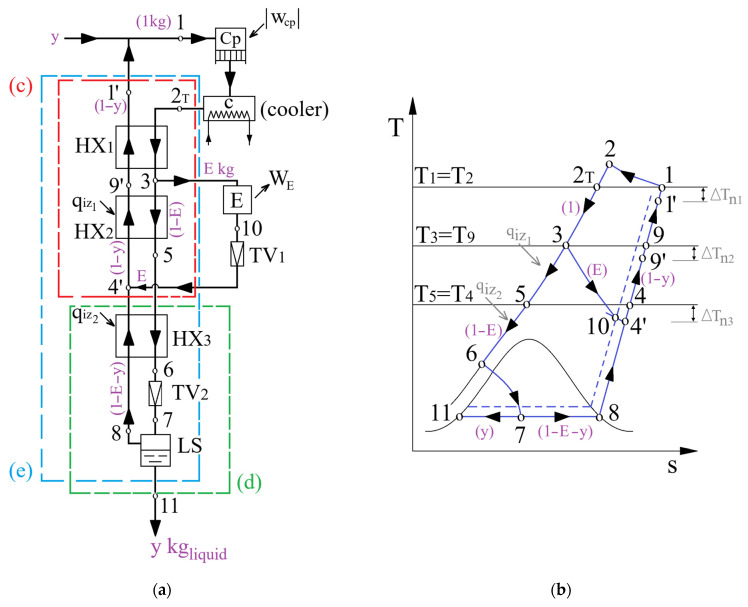
The Claude–Heylandt cycle: (**a**) process flow diagram; (**b**) its corresponding T–s diagram.

The installation scheme and cycle representation in the T–s diagram are shown in Figure 3.

The analysis was carried out for 1 kg of compressed gas (1–2–2T) in compressor Cp and theoretically cooled to the ambient temperature, the suction temperature of the compressor. The resulting high-pressure forward stream is directed to the cryogenic heat exchanger HX_1_, where it transfers heat in counterflow to the low-pressure returning stream, resulting in a temperature decrease to state 3.

In state 3, from the forward stream, fraction E is expanded in an expander to an intermediate pressure p_10_. As a result, mechanical work is produced and a significant decrease in the gas temperature occurs. The remaining main fraction, with mass (1 − E), is further cooled in the heat exchanger HX_2_ and enters in the throttle valve TV_2_ at state 6.

By splitting the forward stream and partially expanding it into an expander, an increase in the heat capacity of the cold returning stream and a decrease in the heat capacity of the hot high-pressure forward stream were obtained simultaneously. Enhancing the imbalance between the heat capacities of the hot forward stream and the cold returning stream leads to a decrease in the temperature of the hot forward stream at the outlet of HX_2_ (state 5).

Following expansion 6–7, in the throttle valve TV_2_, a two-phase mixture is obtained, which in the liquid separator LS is separated into the liquid fraction y, at state 11, and the cold-gas fraction (1–E–y) (state 8), which represents the cold returning stream in HX_3_.

In TV_1_, the amount E of gas expanded in the expander is throttled down to the ambient pressure (10–4′) and mixes with fraction (1–E–y), which represented the cold returning stream from HX_3_.

Subsequently, after passing through HX_3_, the returning stream and the expanded fraction E form the flow (1–y), which enables the cooling of the forward stream in HX_2_.

### 3.2. Mathematical Modeling of the Claude–Heylandt Cryogenic Liquefaction Cycle

To gain a comprehensive thermodynamic understanding, the model integrates conventional energy analysis with exergy analysis, thereby quantifying both the quantity of energy flows and the degradation of their quality within the process.

#### 3.2.1. Energy Analysis of the Claude–Heylandt Cycle

The decision parameters of the Claude–Heylandt cycle are $p_{2}$—the compression pressure, $T_{3}$—temperature at the inlet of the expander, ${\Delta T}_{n1}$–temperature difference at the hot end of HX_1_ and ${\Delta T}_{n3}$—temperature difference at the hot end of HX_3_ (Figure 3b).

As a function of the specified decision parameters, the liquefied mass fraction y, fraction of compressed gas expanded in the expander E, ${\Delta T}_{n2}$—temperature difference at the hot end of HX_2_ and state 6 at the inlet of TV_2_ were calculated.

From Figure 3b it is observed that

(28) $${\Delta T}_{n1}=T_{1}-T_{1^{\prime }}=T_{2T}-T_{1^{\prime }},$$

(29) $${\Delta T}_{n2}=T_{3}-T_{9^{\prime }},$$

(30) $${\Delta T}_{n3}=T_{5}-T_{4^{\prime }}.$$

The layout of the installation (Figure 3a) can be formally divided into two principal zones: the expansion stage in the expander (contour (c)) and throttling stage (contour (d)).

The liquefied gas fraction y results from the energy balance of the global contour (e) (Figure 3a), for 1 kg of compressed gas.

(31) $$h_{2T}+E\cdot h_{4^{\prime }}+q_{iz}=y\cdot h_{11}+E\cdot h_{3}+\left(1-y\right)h_{1^{\prime }}$$

After rearranging the terms, one obtains

(32) $$y\left[\left(h_{1}-h_{11}\right)-\left(h_{1}-h_{1^{\prime }}\right)\right]=h_{1}-h_{2T}+E\left(h_{3}-h_{4^{\prime }}\right)-\left(h_{1}-h_{1^{\prime }}\right)-q_{iz}.$$

The terms in Equation (32) have the following meanings:

$q_{n1}=h_{1}-h_{1^{\prime }}$—represents the cold loss due to inefficiency of HX_1_ in the expansion stage in the expander (contour (c) in Figure 3a), which imposes ${\Delta T}_{n1}$ at the hot end of HX1;

$q_{n3}=h_{4}-h_{4^{\prime }}$—represents the cold loss due to inefficiency of HX_3_, which imposes ${\Delta T}_{n3}$ at the hot end of HX_3_.

$w_{E}=h_{3}-h_{4^{\prime }}=h_{3}-h_{10}$—represents the specific work produced by the expander.

$\left|{\Delta h}_{T_{0}}\right|=h_{1}-h_{2T}$—the isothermal throttling effect at ambient temperature, $T_{1}=T_{0}.$

$q_{iz1}$—the heat ingress on the returning stream during the expander stage.

$q_{iz2}$—the heat ingress on the returning stream during the throttling stage (TV_2_).

$\left|q_{f}\right|=h_{1}-h_{11}$—the specific refrigeration capacity of liquefied gas.

Thus, from Equation (32) one obtains:

(33) $$y=\frac{\left|{\Delta h}_{T_{0}}\right|+E\cdot w_{E}-q_{n1}-q_{iz}}{\left|q_{f}\right|-q_{n1}}.$$

From Equation (33), it is observed that the liquefied gas fraction is imposed by the enthalpy difference in the isothermal compression process at the ambient temperature $\left|{\Delta h}_{T_{0}}\right|$, the mechanical work produced in the expander $w_{E}=\Delta h_{E},$ the fraction E of gas expanded in the expander and the cold loss $q_{L}=q_{n1}+q_{iz}$.

In this context, we expect that the cooling of the gas in the expander recovers some of the cold losses.

For the selected decision parameters $\left(p_{2},\;T_{3},\;{\Delta T}_{n1},\;{\Delta T}_{n3}\right)$ and for the fixed parameters $p_{1}$ and $T_{1}=T_{2T}=T_{0}$, the dependent parameters y and E were calculated by solving the energy balance equations for the two cooling stages of the system: the expansion stage in the expander and the throttling stage (TV_2_).

For the throttling stage (contour (d) in Figure 3a), the energy balance for 1 kg of compressed gas is

(34) $$\left(1-E\right)h_{5}+q_{iz}=y\cdot h_{11}+\left(1-E-y\right)h_{4^{\prime }}.$$

which finally leads to

(35) $$\left(1-E\right)\left(h_{4}-h_{5}\right)-\left(1-E\right)\left(h_{4}-h_{4^{\prime }}\right)-q_{iz2}=y\left[\left(h_{4}-h_{11}\right)-h_{4}-h_{4^{\prime }}\right],$$

(36) $$\left(1-E\right)\left|{\Delta h}_{T_{5}}\right|-\left(1-E\right)q_{n3}-q_{iz2}=y\left(\left|q_{f}\right|-q_{n3}\right).$$

It is observed that the values of the state variables, and therefore the values of $\left|{\Delta h}_{T_{5}}\right|$, $q_{n3}$ and $\left|q_{f}\right|$, do not change with the variation in fraction E (Equation (36)).

An increase in fraction E of the gas expanded in the expander reduces both the isothermal throttling effect at temperature $T_{5}$ ($\left(1-E\right)\left|{\Delta h}_{T_{5}}\right|)$ and the cold loss $\left(1-E\right)q_{n3})$ caused by the inefficiency of the recuperative heat exchanger of the throttling stage.

The two effects, having opposite directions, impose different trends in the variation in the liquefied gas fraction y as fraction E is modified.

The second equation, which, together with Equation (36), forms the system used to calculate the dependent variables E and y, is the energy balance of the expansion stage of the gas in the expander (contour (c) in Figure 3a):

(37) $$h_{2T}+E\cdot h_{4^{\prime }}+q_{iz1}+\left(1-E-y\right)h_{4^{\prime }}=E\cdot h_{3}+\left(1-E\right)h_{5}+\left(1-y\right)h_{1^{\prime }}.$$

Equation (37) can be rewritten as follows:

(38) $${(h}_{1^{\prime }}-h_{2T})+E(h_{3}-h_{5})+h_{5}-h_{4^{\prime }}-q_{iz1}=y(h_{1^{\prime }}-h_{4^{\prime }}).$$

To highlight the cold losses due to the inefficiency of the heat exchangers, resulting from the temperature differences at the hot end ${\Delta T}_{n1}$ and ${\Delta T}_{n2}$, Equation (38) can be written as

(39) $$\left|{\Delta h}_{T_{0}}\right|+E(h_{3}-h_{5})-\left|{\Delta h}_{T_{5}}\right|+q_{n2}-q_{n1}-q_{iz1}=y[{(h}_{1}-h_{4})-q_{n1}+q_{n2}].$$

#### 3.2.2. Pinch Analysis of the Linde–Hampson Cryogenic Cycle with Claude–Heylandt Modification

Based on the Pinch analysis, in which the zero-utility condition was imposed, the hot and cold composite curves of the second recuperative heat exchanger HX_2_ are represented. Compared to the Linde cycle, the novelty of this heat exchanger is the pronounced imbalance between the heat capacities of the hot and returning stream in the region where the gas undergoes expansion in the expander.

**Figure 4:**
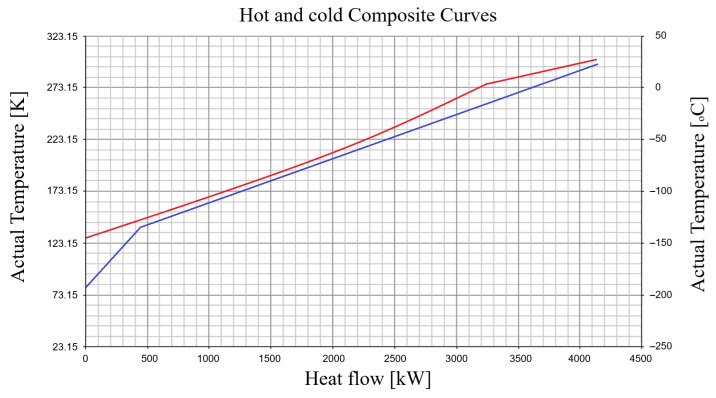
The second recuperative heat exchanger HX_2_ of the expansion stage in the expander within the Claude–Heylandt-type air liquefaction scheme—temperature profiles of the hot and returning composite streams against heat exchange or the corresponding heat transfer surface.

For the specified operating conditions ($p_{1}=0.1\;MPa$, $p_{2}=16\;MPa$, $\Delta T_{n1}=4\;K$, $\Delta T_{n3}=8\;K$, $T_{3}=276\;K$), the temperature difference at the Pinch is $\Delta T_{P}=4\;K$, occurring at the Pinch temperature $T_{P}=193.15\;K$ ($t_{P}=-80\;{}^{\circ}C$) (Figure 4).

#### 3.2.3. Exergy-Based Analysis of the Claude–Heylandt Cycle

Applying the steady-flow exergy balance to the global contour (e) in Figure 3a yields

(40) $$\sum {Ex}_{Q}=\Delta Ex+\sum W+\sum I_{i}.$$

By inspecting the terms in Equation (40), along the contour (c), it is observed that its boundaries are crossed only by the substance and heat streams.

The terms of the exergy balance equation have the following composition:

(41) $$\sum {Ex}_{Q}=\sum {Ex}_{Qiz}={Ex}_{Q_{iz1}}+{Ex}_{Q_{iz2}}.$$

It is considered that the heat ingress from the surroundings occurs at the level of the returning stream in HX_2_ and HX_3_. Their rejection toward the ambient environment represents losses for the system.

For example, the exergy transfer rate accompanying the undesired heat gain from the environment through the incomplete insulation of heat exchanger HX_2_ is

(42) $${Ex}_{Q_{iz1}}=Q_{iz1}\left(1-\frac{T_{0}}{{T_{M}}_{4^{\prime }-9^{\prime }}}\right)<0,$$

where

$T_{M_{4^{\prime }-9^{\prime }}}=\frac{h_{9^{\prime }}-h_{4^{\prime }}}{s_{9^{\prime }}-s_{4^{\prime }}}$ is the mean thermodynamic temperature between states 9′ and 4′ in the cycle.

$Q_{iz1}>0$ is the heat received from the surroundings.

$T_{M_{4^{\prime }-9^{\prime }}}<T_{0}$—the mean thermodynamic temperature between states 9′ and 4′ of the cycle was lower than the ambient temperature.

The exergy of the heat ingress through the insulation is negative and represents the minimum mechanical work that would need to be supplied to reject it to the ambient environment. Therefore, there is an evident loss for the system:

(43) $${Ex}_{Q_{iz1}}=-\left|{Ex}_{Q_{iz1}}\right|=-L_{Q_{iz1}}.$$

Similarly,

(44) $${Ex}_{Q_{iz2}}=Q_{iz2}\left(1-\frac{T_{0}}{{T_{M}}_{8-4^{\prime }}}\right)<0\;and$$

(45) $${Ex}_{Q_{iz2}}=-\left|{Ex}_{Q_{iz2}}\right|=-L_{Q_{iz2}}.$$

For the two heat ingress contributions from the surroundings, one obtains

(46) $$\sum {Ex}_{Qiz}=-L_{Q_{iz1}}-L_{Q_{iz2}}={-L}_{Q_{iz}}.$$

The exergy variation in the substance streams becomes

(47) $$\Delta Ex={Ex}_{3}+{Ex}_{11}+{Ex}_{1^{\prime }}-{ex}_{2T}-{Ex}_{4^{\prime }}$$

It is observed that state 2T is located at ${(p}_{2T}>p_{1}=p_{0})$, and its exergy represents the absolute value of the mechanical work of isothermal compression at $T_{0}$, from the ambient pressure $p_{0}$ to $p_{2}$. Thus,

(48) $${ex}_{2T}=\left|w_{cp,T_{0}}\right|.$$

It is observed that

(49) $${Ex}_{11}=y\left|{ex}_{q_{f}}\right|=\left|{Ex}_{Q_{f}}\right|.$$

(50) $${Ex}_{1^{\prime }}=\left(1-y\right)\left[h_{1^{\prime }}-h_{1}-T_{0}\left(s_{1^{\prime }}-s_{1}\right)\right]=L_{{\Delta T}_{n1}},$$

represents the exergy loss of the heat that cannot be recovered from the forward stream, due to the requirement to maintain a temperature difference (ΔT_n1_) at the hot end of the recuperative heat exchanger HX_1_.

(51) $${\mathrm{Ex}}_{3}-{\mathrm{Ex}}_{4^{\prime }}=E\left[\left(h_{3}-h_{4^{\prime }}\right)-T_{0}\left(s_{3}-s_{4^{\prime }}\right)\right]={E\left[\left(h_{3}-h_{10}\right)+T_{0}\left(s_{10}-s_{3}\right)+T_{0}(s_{4^{\prime }}-s_{10})\right]=W}_{E}+I_{E}+I_{t_{2}}.$$

By substituting Equations (46) and (48)–(51) into Equation (40), we obtain

(52) $$\left|w_{cp,T_{0}}\right|=\left|{Ex}_{Q_{f}}\right|+W_{E}+I_{E}+I_{t_{1}}+{I_{t_{2}}+I}_{\Delta T1}+I_{\Delta T2}+I_{\Delta T3}+L_{{\Delta T}_{n1}}+L_{Q_{iz}},$$

The exergy destruction was determined using the Gouy–Stodola relation [33,34].

(53) $$I_{t1}=\left(1-E\right)T_{0}\left(s_{7}-s_{6}\right),$$

(54) $$I_{t2}=E\cdot T_{0}\left(s_{4^{\prime }}-s_{10}\right),$$

(55) $$I_{\Delta T1}=T_{0}\left[\left(1-y\right)s_{1^{\prime }}+s_{3}-s_{2T}-\left(1-y\right)s_{9^{\prime }}\right]$$

(56) $$I_{\Delta T2}=T_{0}\left[\left(1-E\right)s_{5}+\left(1-y\right)s_{9^{\prime }}-\left(1-E\right)s_{3}-\left(1-y\right)s_{4^{\prime }}\right],$$

(57) $$I_{\Delta T3}=T_{0}\left[\left(1-E\right)s_{6}+\left(1-E-y\right)s_{4^{\prime }}-\left(1-E\right)s_{5}-\left(1-E-y\right)s_{8}\right].$$

Considering the isentropic efficiency of the compression process, the exergy balance Equation (52) becomes

(58) $$\left|w_{cp,T_{0}}\right|-W_{E}=\left|{Ex}_{Q_{f}}\right|+I_{E}+{I_{cp}+I}_{t_{1}}+{I_{t_{2}}+I}_{\Delta T1}+I_{\Delta T2}+I_{\Delta T3}+L_{{\Delta T}_{n1}}+L_{Q_{iz}},$$

in which

(59) $$I_{cp}=\left|w_{cp}\right|-\left|w_{cp,T_{0}}\right|=\left|w_{cp,T_{0}}\right|\left(\frac{\left|w_{cp,T_{0}}\right|}{\eta_{T,cp}}-1\right).$$

Under the conditions in which the mechanical work produced by the expander is recovered, the exergetic efficiency of the system and the share of a destruction or loss of mechanical work in the total mechanical work consumption of the system are as follows:

(60) $$\eta_{ex}=\frac{\left|{Ex}_{Q_{f}}\right|}{\left|w_{cp}\right|-W_{E}}\;,\;and$$

(61) $$\Psi =\frac{I_{i},L_{i}}{\left|w_{cp}\right|-W_{E}}.$$

### 3.3. Energy and Exergy Analysis Results of the Claude–Heylandt Cryogenic Cycle

The analysis of the system operation under variations in the decision parameters ${\Delta T}_{n1},\;{\Delta T}_{n3},\;T_{3}$ and $p_{2}$ was performed under the conditions $p_{1}=0.1\;MPa$, $T_{1}=300\;K$ and $\eta_{T,cp}=0.6$.

The mathematical model incorporates several key assumptions necessary for a consistent comparative exergy analysis. The compressor isentropic efficiency was set to 0.6. Although modern compressors can achieve higher efficiencies in practical applications, this value is deliberately maintained for two primary reasons. First, it ensures direct comparability with the results of our previous exergetic study on Linde–Hampson cycles [7], in which the same assumption was used. Second, constant and moderate efficiency is a common and accepted simplification in foundational thermodynamic models, focusing on the comparative performance of cycle configurations rather than on the absolute performance of state-of-the-art machinery. This approach isolates the impact of the cycle layout and fluid properties on exergy destruction from the variable performance of the specific equipment.

**Figure 5:**
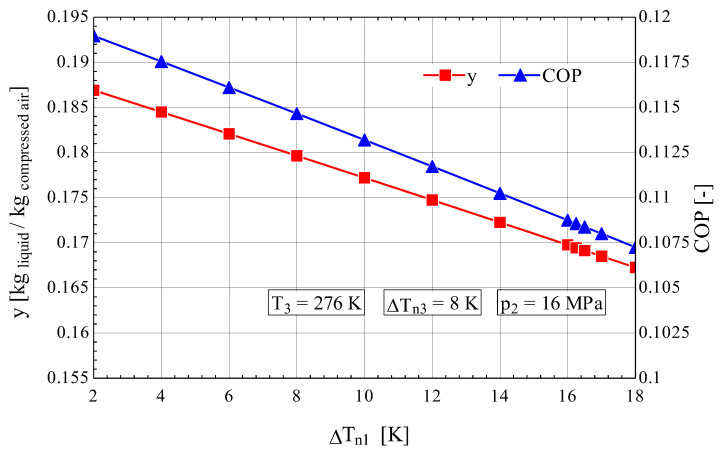
Claude–Heylandt cryogenic cycle. Liquefied air fraction y and cycle coefficient of performance against $\Delta T_{n1}$ at the hot end of HX_1_.

**Figure 6:**
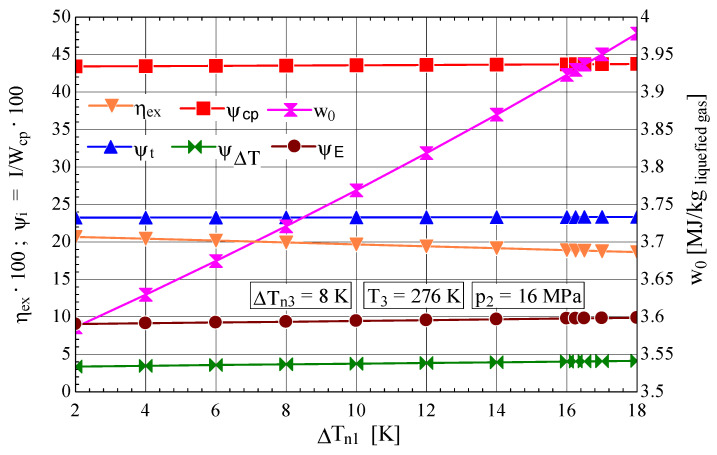
Claude–Heylandt cryogenic cycle. Shares of exergy destruction, $\eta_{ex}$, and $w_{0}$ per kilogram of liquefied air against $\Delta T_{n1}$ at the hot end of HX_1_.

It is observed that an increase in ${\Delta T}_{n1}$ generates, similarly to the case of Linde cryogenic installations with one throttling stage or with two throttling stages, a rapid decrease in the liquefied gas fraction y and in the cycle coefficient of performance (Figure 5). The mechanical work consumption for 1 kg of liquefied gas exhibited a pronounced increase as y decreased with an increase in ${\Delta T}_{n1}$ (Figure 6).

It is noted that, under the same operating conditions ($p_{2}=16\;MPa$ și ${\Delta T}_{n1}=4\;K$), as in the case of Linde installations with one or two throttling stages [7], the Claude installation exhibits a significant increase in both the liquefied fraction y ($y_{L,1t}=0.0505$, $y_{L,2t}=0.043$ and $y_{C}=0.1845$), and the coefficient of performance (${COP}_{L,1t}=0.02933$, ${COP}_{L,2t}=0.0446$ and ${COP}_{C}=0.1145$), as well as a considerable decrease in the mechanical work consumption ($w_{0,L1t}=14.41,\;w_{0,L2t}=9.49$ and $w_{0,C}=3.63$) [MJ/(kg liquefied air)] (Figure 5 and Figure 6).

By introducing an additional cold contribution into the returning stream, obtained by diverting a fraction of gas from the forward stream toward the expander, so that it absorbs a larger amount of heat from the forward stream by reducing the flow rate as a result of this stream splitting, an improvement in the exergetic performance of the process has been achieved.

Thus, compared with the Linde cycles with one or two throttling stages [7], in the case of the installation operating according to the Claude cycle, the share of exergy destruction in the throttle valve $\Psi_{t}$ ($\Psi_{t,L1t}=41.15\%$, $\Psi_{t,L2t}=29.49\%$, $\Psi_{t,C}=23.44\%$) and that caused by heat transfer at a finite temperature difference ΔT in the heat exchanger ($\Psi_{\Delta T,L1t}=13.89\%,\;\Psi_{\Delta T,L2t}=16.89\%$ and $\Psi_{\Delta T,C}=3.471\%$) decreased considerably, whereas the exergetic efficiency of the system improved significantly ($\eta_{ex,L1t}=5.1\%;\;\eta_{ex,L2t}=7.8\%$ and $\eta_{ex,C}=20.4\%$).

To support the validity of the proposed mathematical model, a quantitative comparison with peer-reviewed performance data for the Linde–Hampson and Claude-type air-liquefaction cycles is reported in Appendix A (Table A1). The present results reproduce both the established performance hierarchy (higher liquefaction yield and exergetic efficiency for Claude–Heylandt relative to Linde–Hampson) and the expected order of magnitude of the key indicators, confirming that the observed superiority is physically consistent rather than a modeling artifact.

**Figure 7:**
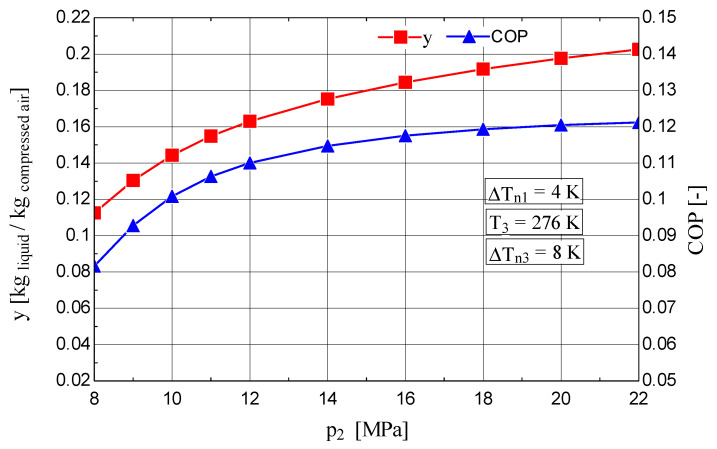
Claude–Heylandt cryogenic cycle. Liquefied air fraction y and cycle coefficient of performance as functions of the compression pressure $p_{2}$.

**Figure 8:**
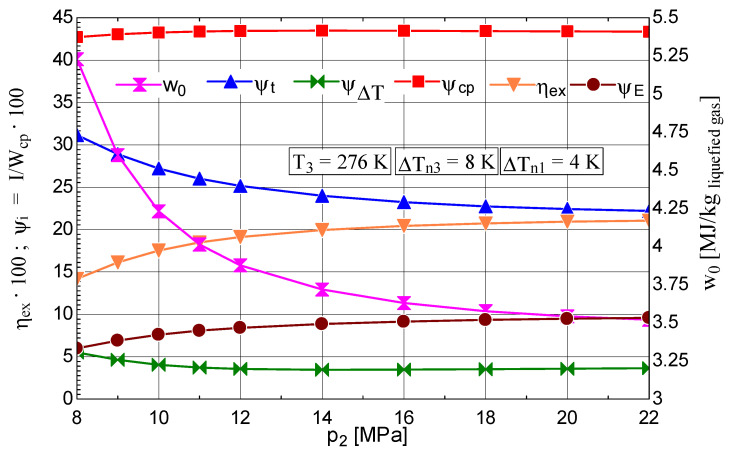
Claude–Heylandt cryogenic cycle. Shares of exergy destruction, $\eta_{ex}$, and $w_{0}$ per kilogram of liquefied air against $p_{2}$.

An increase in the compression pressure ($p_{2}$) led to higher values of the liquid fraction y and coefficient of performance (Figure 7) and a decrease in $w_{0}$ (Figure 8).

The reduction in the shares of exergy destruction $\Psi_{\Delta T}$, $\Psi_{t}$ and $\Psi_{E}$ in the mechanical work consumption of the compressor resulted in an increase in $\eta_{ex}$ (Figure 8).

**Figure 9:**
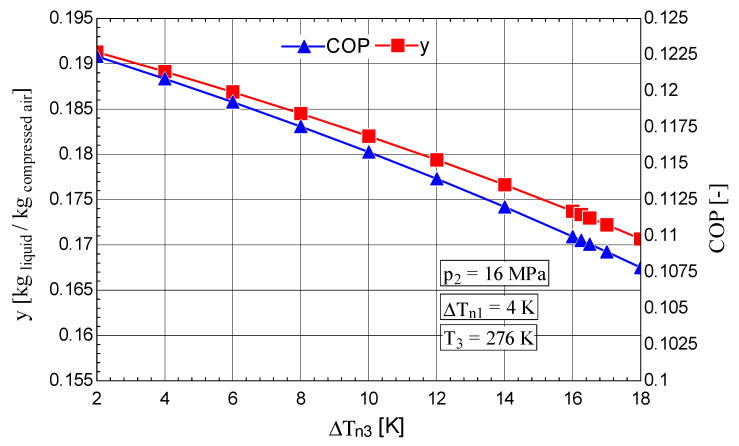
Claude–Heylandt cryogenic cycle. Liquefied air fraction y and the cycle coefficient of performance against $\Delta T_{n3}$ at the hot end of HX_3_.

**Figure 10:**
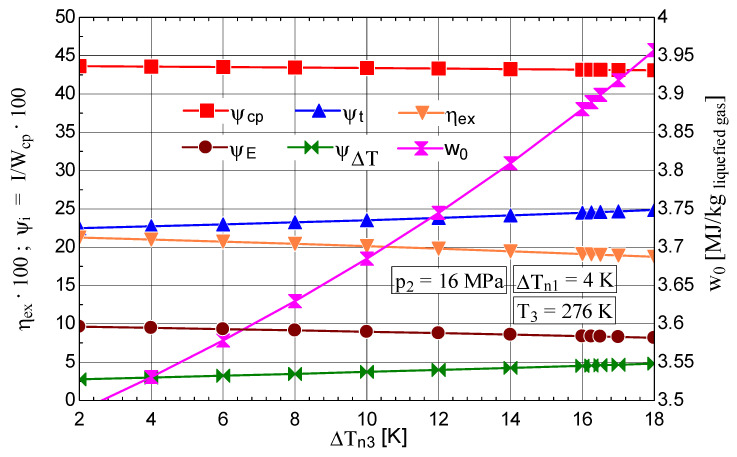
Claude–Heylandt cryogenic cycle. The shares of exergy destruction, $\eta_{\mathrm{ex}}$, and $w_{0}$ per kilogram of liquefied air against $\Delta T_{n3}$ at the hot end of HX_3_.

The increase in the temperature difference $\Delta T_{n3}$ at the hot end of HX_3_ of the throttling stage causes a rapid decrease in the fraction of liquefied gas y and the coefficient of performance of the cycle (Figure 9), concomitantly with an increase in the mechanical work consumption per 1 kg of liquefied gas (Figure 10).

The increase in the shares of exergy destruction $\Psi_{\Delta T}$ and $\Psi_{t}$ in the mechanical work consumption of the compressor led to a decrease in $\eta_{ex}$ (Figure 10).

**Figure 11:**
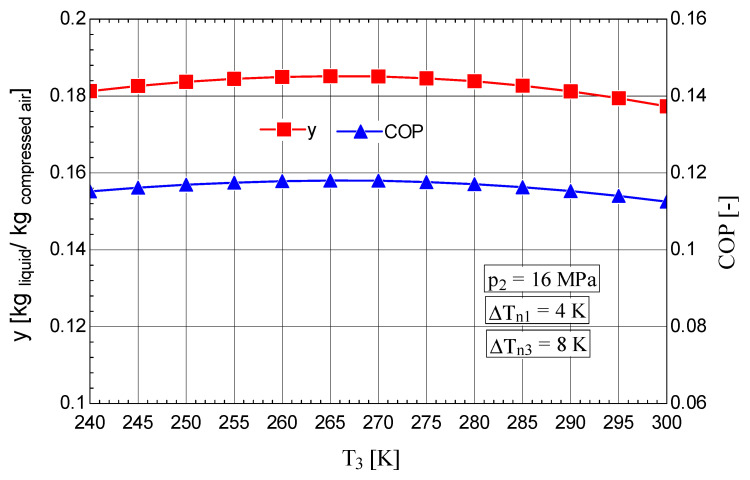
Claude–Heylandt cryogenic cycle. Liquefied air fraction y and the cycle coefficient of performance against $T_{3}$ at the inlet of the expander.

**Figure 12:**
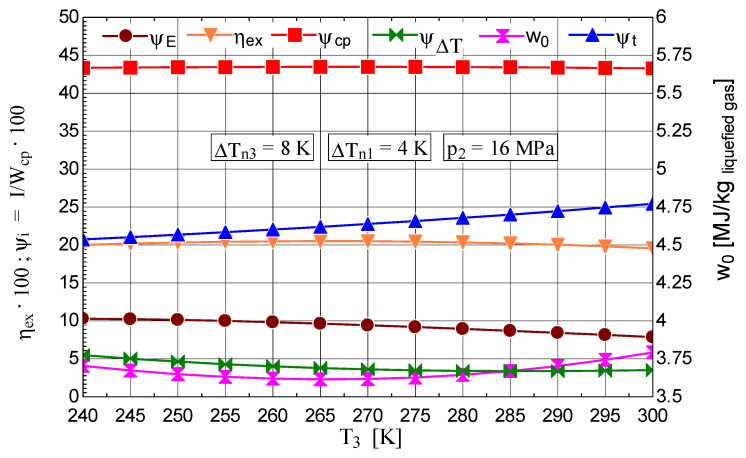
Claude–Heylandt cryogenic cycle. The shares of exergy destruction, $\eta_{ex}$, and $w_{0}$ per kilogram of liquefied air against $T_{3}$ at the inlet of the gas into the expander.

The analysis of the variation in decision parameter $T_{3}$ allowed the determination of the optimal operating regime from the perspective of the inlet temperature into the expander, both qualitatively and quantitatively. The results showed that for the value $T_{3}=265\;K$, a maximum liquefied fraction y=0.1852 is reached (Figure 11), concomitantly with a minimum mechanical work consumption per 1 kg of liquefied gas $w_{0}=3.616\left[\frac{MJ}{kg\;liquefied\;gas}\right]$ and a maximum exergetic efficiency $\eta_{\mathrm{ex}}=0.2051$ (Figure 12).

From the analysis of the parameters, the positive effect of reducing the gas temperature at the inlet to the throttle valve is highlighted by the decrease in the share of exergy destruction associated with the throttling process (Figure 12).

Comparative energetic and exergetic analyses performed on the cryogenic gas liquefaction cycles—the Linde–Hampson cycle with a single throttling, the Linde cycle with two throttling stages [7] and the Claude–Heylandt cycle—allowed the identification of the processes and equipment with the highest shares of exergy destruction, providing a solid basis for optimizing the overall performance. The results highlight a progressive reduction in the share of the exergy destroyed in the throttle valve ($\psi_{t,L1t}=41.15\%;$ $\psi_{t,L2t}=29.49\%$ and $\psi_{t,C}=23.44\%$) and in the heat exchanger, owing to heat transfer at a finite temperature difference ($\psi_{\Delta T,L1t}=13.89\%$, $\psi_{\Delta T,L2t}=16.89\%$ and $\psi_{\Delta T,C}=3.47\%$), in parallel with an increase in the exergetic efficiency ($\eta_{ex,L1t}=5.1\%$, $\eta_{ex,L2t}=7.8\%$ and $\eta_{ex,C}=20.4\%$).

Based on these results, the next stage of this study aims to extend the use of the Claude cycle by integrating a rectification column intended for oxygen separation. The objective is to perform an exergetic evaluation of the possible coupling solutions and identify the configuration that ensures the maximum efficiency of the system. Simulations were performed using CAPE-OPEN to CAPE-OPEN ChemSep [35,36].

## 4. Integration of a Rectification Column into the Claude–Heylandt Cryogenic Cycle and Its Optimization Based on the Exergy Analysis

This study proposes an analysis of the integration of a rectification column into the Claude–Heylandt cryogenic cycle and the optimization of its operation based on exergy analysis. The objective of this research is to identify the optimal configuration of the column, considering the essential decision parameters, namely, the number of trays and the position of the liquid-air feed point of the column.

To reduce resource consumption in the rectification column (RC), the analysis focuses on limiting the exergy destruction generated by irreversible processes within the column. The determination of the exergy consumption will be carried out by applying the entropic balance to the column, and the generated entropy will be calculated as the difference between the outlet and inlet entropy streams and those associated with the thermal energy exchanges with the external environment. The air separation (rectification) column was calculated using ChemSep software v8.46 [36].

### 4.1. Functional Description of the System

The analyzed case concerns the integration of a rectification column into the Claude–Heylandt cryogenic cycle, which operates with the parameters $p_{1}=0.1\;MPa$, $p_{2}=16\;MPa$, $\Delta T_{n1}=4\;K$, $\Delta T_{n3}=8\;K$, $T_{3}=276\;K$. The column was fed with liquid air at a flow rate of y = 0.185 [kg/kg of compressed air]. The objective was to optimize the operation of the rectification column by selecting the number of trays and vertical position of the liquid-air feed point in the column, with the aim of obtaining liquid oxygen with purity above 99%.

**Figure 13:**
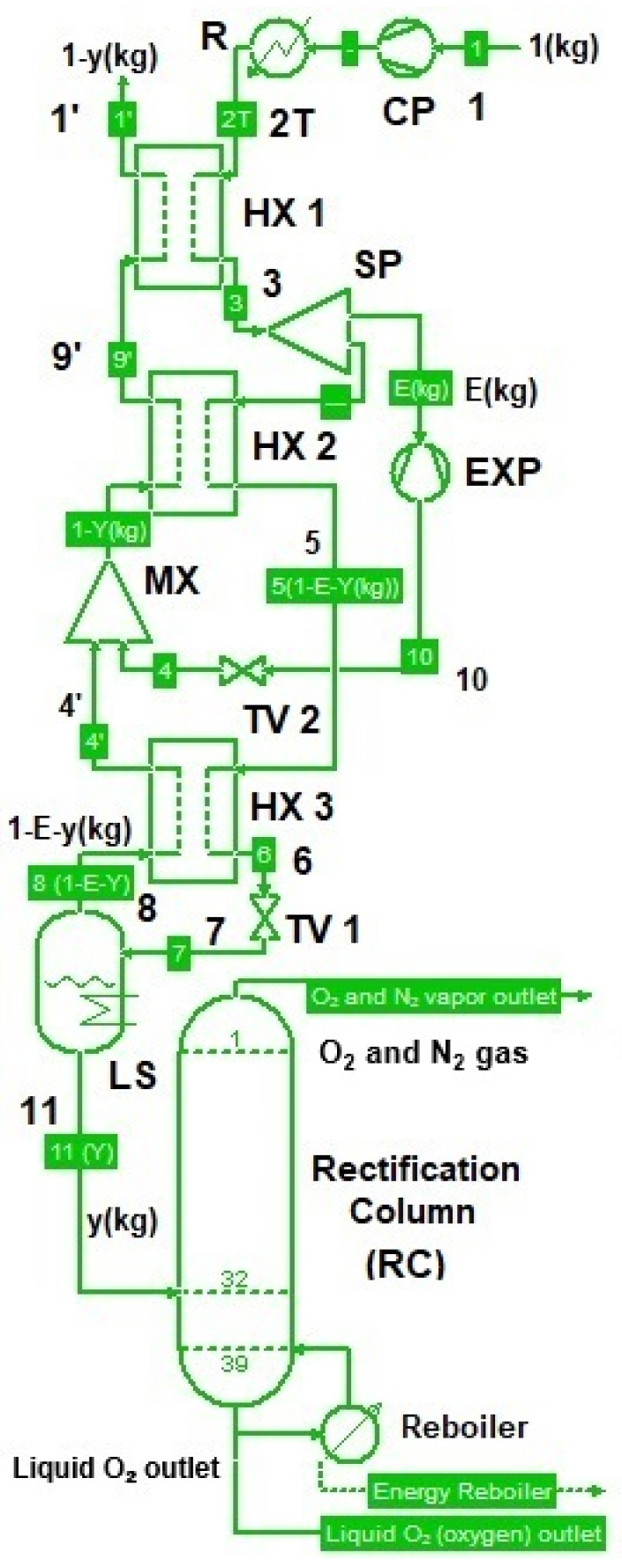
Schematic of the Claude–Heylandt cryogenic liquefaction cycle with a rectification column for the separation of O_2_ from air.

The schematic of the oxygen separation unit is presented in Figure 13.

The liquefied air (y = 0.185 kg/kg of compressed air), obtained using the Claude–Heylandt cycle, was introduced into the rectification column. In this column, the separation of the main components, nitrogen and oxygen, occurred because of the differences in their vaporization temperatures.

In the lower part of the column, oxygen accumulated in the liquid state, as it had a higher vaporization temperature than nitrogen, while in the upper part, a gaseous mixture with a high nitrogen concentration was collected.

At the bottom of the column, the reboiler provides the thermal energy required for the vaporization of the nitrogen remaining in the oxygen-rich solution. The vapor mixture, which is rich in nitrogen, moves upward through the column, whereas the liquid stream flows downward, owing to gravity. On each tray of the column, a heat and mass transfer process occurred between the vapor and the liquid. Nitrogen is more volatile and vaporizes, thereby enriching the descending liquid stream with oxygen. At the top of the column, a gaseous mixture with a high nitrogen concentration was obtained, whereas at the bottom, high-purity liquid oxygen was obtained.

### 4.2. Mathematical Model of the Separation Process

The mathematical model was based on exergy analysis of the separation column. The analysis of the irreversibilities of the gas separation processes in the rectification column focuses on the entropy generation associated with the process. The entropy values corresponding to the inlet and outlet states of the substance streams in the rectification column were obtained from simulations performed using ChemSep software.

#### 4.2.1. Exergy Analysis

**Figure 14:**
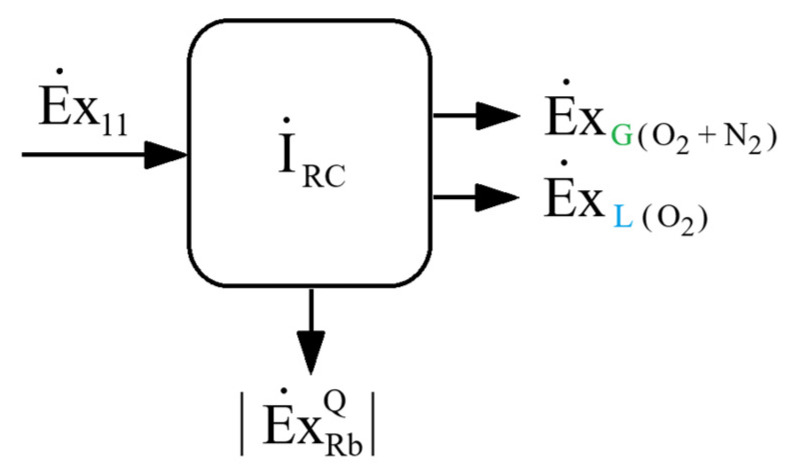
Schematic of the interaction with the external environment for the rectification column in the air separation unit based on the Claude–Heylandt cycle.

The exergy interactions of the rectification column with the external environment, corresponding to the configuration presented in Figure 13, are schematically illustrated in Figure 14.

Heat transfer to the separation column through the reboiler occurs at a temperature lower than that of the external environment, which results in a negative exergy associated with this thermal flux. This justifies the representation of the absolute value of heat exergy in the reboiler, as shown in Figure 14,

(62) $${\dot{Ex}}_{Rb}^{Q}=-\left|{\dot{Ex}}_{Rb}^{Q}\right|.$$

The exergy destruction is

(63) $${\dot{I}}_{RC}=T_{0}\cdot {\dot{S}}_{gen}^{RC}.$$

From the entropic balance

(64) $${\dot{S}}_{gen}^{RC}=\sum {\dot{S}}_{e}-\sum {\dot{S}}_{i}-\sum \frac{\dot{Q}}{T},$$

applied to the schematic in Figure 14, one obtains

(65) $${\dot{S}}_{gen}^{RC}=m_{G(O_{2}+N_{2})}\cdot s_{G\left(O_{2}+N_{2}\right)}+m_{L\left(o_{2}\right)}\cdot s_{L\left(o_{2}\right)}-y\cdot s_{11}-\frac{{\dot{Q}}_{Rb}}{T_{Rb}}.$$

The share of exergy destruction in the rectification column, in the mechanical work consumption of the installation, is

(66) $$\Psi_{RC}=\frac{{\dot{I}}_{RC}}{\left|{\dot{w}}_{cp}\right|-{\dot{W}}_{E}}.$$

#### 4.2.2. Results of Exergy Analysis for Optimization of Rectification Column Configuration Attached to Claude–Heylandt Cycle

The decision parameters were the number of trays and the position of the liquid-air feed point in the column. The reference ambient environment is characterized by $p_{1}=0.1\;MPa$ and $T_{0}=300\;K.$

This study examines how the variation in the number of trays and the position of the liquid-air feed point in the column influences the exergy destruction in the separation column.

**Figure 15:**
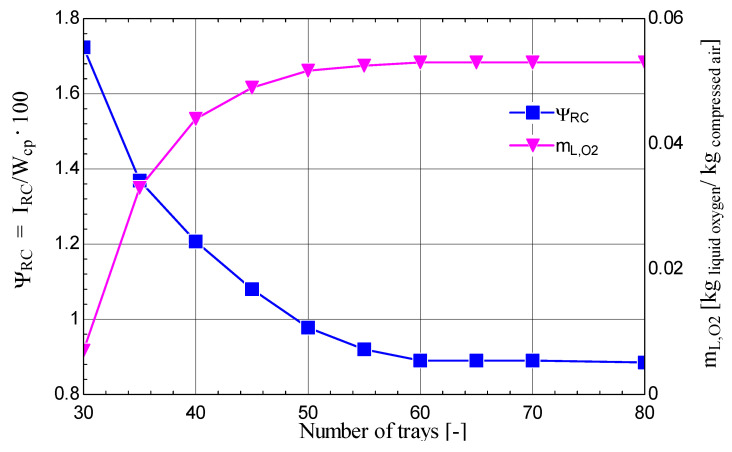
Share of the exergy destruction and the oxygen flow rate (with 99% purity) extracted from the cryogenic rectification column as a function of the number of trays.

Thus, the influence of the number of trays on the share of the exergy destruction in the cryogenic rectification column attached to the Claude–Heylandt cycle, as well as on the extracted flow rate of liquid oxygen with 99% purity, can be observed in Figure 15. It was observed that increasing the number of trays led to a rapid decrease in the exergy destruction in the mechanical work consumption and to an increase in the extracted flow rate of liquid oxygen with 99% purity. It is noted that, at a certain point, a maximum threshold of efficiency is reached, beyond which increasing the number of trays no longer improves the performance, and thus, the optimization limit is reached. Therefore, for the cryogenic rectification column, there is a minimum value of the number of trays (60) for which the share of the exergy destruction is minimal (0.89%) and the oxygen flow rate (with 99% purity) is maximal.

**Figure 16:**
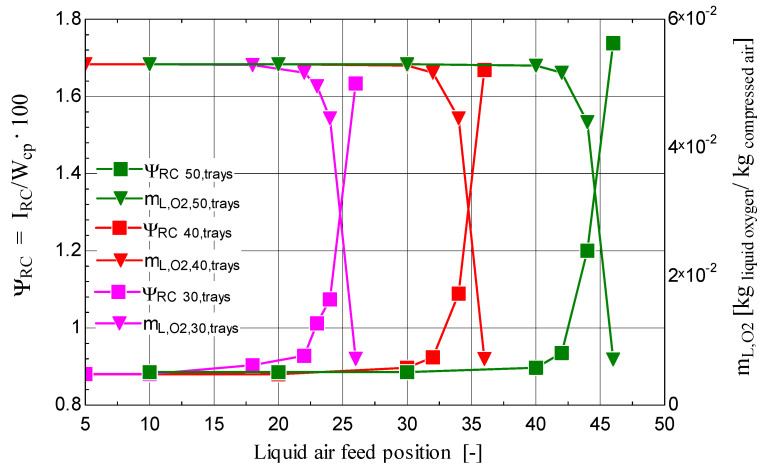
Share of the exergy destruction and the oxygen flow rate (with 99% purity) extracted from the cryogenic rectification column as functions of the number of trays and of the position, relative to the top end of the column, of the tray corresponding to the liquid-air feed point.

The influence of the position of the liquid-air feed point on the share of exergy destruction in the cryogenic rectification column attached to the Claude–Heylandt cycle, and implicitly on the exergy destruction, is shown in Figure 16. The exergy destruction is minimal when liquid air is introduced at the upper part of the column; then, as the elevation of the feed point decreases, the share of exergy destruction increases slightly, relative to the high electric-energy consumption of the system. Feeding the rectification column at the lower part leads to a very rapid increase in the exergy destruction associated with the separation process (Figure 16).

## 5. Conclusions

The recuperative heat exchanger of the cryogenic gas-liquefaction installation extracts heat from the high-pressure forward stream and transfers it to the external environment through the cold returning stream. The extraction and transfer of heat require a consumption of mechanical energy.

The minimization of exergy consumption caused by the irreversibility of heat transfer at a finite temperature difference in the recuperative heat exchanger can be achieved by decreasing the temperature difference between the two streams as the heat-exchange process approaches the cold end of the apparatus. For this purpose, an imbalance between the heat capacities of the two heat-carrying streams was introduced by extracting a fraction of the flow rate of the hot forward stream at a high pressure, expanding it in an expander arranged in parallel, and feeding this cold fraction into the low-pressure cold returning stream.

For the newly developed Claude–Heylandt-type cycle, the share of exergy destruction in the heat exchanger decreased from 14% in the case of the Linde–Hampson cycle with a single throttling to 3.5%.

The exergy analysis applied to the Claude–Heylandt air-liquefaction cycle highlights the manner in which the exergy introduced into the system through the mechanical compression stage is consumed to cover the irreversibility of the working processes and the losses imposed by the efficiency of the recuperative heat exchangers, which is in turn constrained by the necessity of ensuring a temperature difference at the hot end of these heat exchangers.

The increase in the temperature differences $\Delta T_{n1}$ and $\Delta T_{n3}$ at the hot ends of the first and third recuperative heat exchangers has the negative effect of increasing the shares of exergy consumption in the apparatus $\Psi_{\Delta T}$, in the throttle valve $\Psi_{t}$, and in reducing the liquefied-air fraction y. An important effect is associated with $\Delta T_{n3}$, whose doubling leads to a 3.5-fold increase in the exergy destruction share $\Psi_{\Delta T}$.

The increase in the compression pressure $p_{2}$ has a beneficial effect, leading to an increase in the liquefied-air fraction y and a decrease in the compression mechanical work consumed per 1 kg of liquefied air. The reason for the more efficient operation of the cryogenic liquefaction cycle is the reduction, with increasing pressure, in the share of exergy destruction in the throttling process of the throttling stage $\Psi_{t}$.

Compared to the Linde installation with one or two throttling stages, the exergetic efficiency in the case of the Claude cycle increased four times ($\eta_{ex,L1t}=5.1\%;$ and $\eta_{ex,C}=20.4\%$).

In this study, the integration of a cryogenic rectification column into the Claude–Heylandt cycle was analyzed to optimize it based on exergy analysis. The obtained results allowed a quantitative and qualitative evaluation of the process, both in terms of exergy destruction and performance related to the flow rate of liquid oxygen with 99% purity. The analysis highlighted the areas with major dysfunctions and allowed identification of the optimal operating points and configurations of the column.

Through the analysis of the influence of the number of trays, it was found that, as this number increased, the exergy destruction relative to the mechanical-work consumption decreased, while the flow rate of liquid oxygen with 99% purity increased (Figure 16). Regarding the position of the liquid-air feeding point, the results showed that the exergy destruction decreased as this point was placed closer to the upper part of the rectification column (Figure 16).

Thus, the study demonstrated that exergy analysis is an essential tool for optimizing the configuration of the rectification column and in identifying the operating parameters that ensure an optimal balance between energetic efficiency and process performance.

The credibility of the proposed model is additionally supported by a quantitative validation against published performance ranges for Linde–Hampson and Claude-type cycles, as summarized in Appendix A (Table A1).

The present study is based on steady-state thermodynamic and exergetic analysis, with idealized representations of certain components and constant efficiencies. Although this approach is suitable for identifying fundamental trends and optimal structural configurations, it does not capture transient operation, control strategies, or detailed mechanical design aspects. Future work will focus on extending the analysis to dynamic operation, exergoeconomic optimization, and the integration of high-fidelity component models. Experimental validation and industrial-scale case studies are also envisaged as important directions for future research.

## Figures and Tables

**Table 1:** Single stage Linde–Hampson cycle—functional characteristics.

${\Delta T}_{n}$	$p_{1}$	$p_{2}$	y	COP	$\Psi_{\Delta T}$	$w_{0}$	$\eta_{\mathrm{Ex}}$
[K]	[MPa]	[MPa]	$\left[\frac{{kg}_{liquid}}{{kg}_{compressed\;air}}\right]$	[-]	[%]	$\left[\frac{MJ}{{kg}_{liquid\;air}}\right]$	[%]
4	0.1	16	0.05057	0.02933	13.89	14.41	5.148

## Data Availability

Data are contained within the article.

## Abbreviations

The following abbreviations are used in this manuscript:

A	heat exchange area, m^2^
ARS	Solar Absorption System
ASU	Air Separation Unit
C	cooler
COP	Coefficient of Performance
CP	compressor
E	Expander
HX	Recuperative Heat Exchanger
L	Lagrangian
LNG	Liquefied Natural Gas
LS	Liquid separator
MRC	Mixed-Refrigerant Cycle
MX	Mixer
ORC	Organic Rankine Cycle
RC	Rectification Column
SMR	Steam Methane Reforming
SP	Splitter
TV	Throttling Valve
Lattin Letters	
$c_{p}$	specific heat at constant pressure, kJ/kg/K
E	fraction of gas expanded in the expander, kg expanded gas/kg compressed gas
Ex	exergy, kJ
e, ex	mass exergy, kJ/kg
f	objective-function integrand
g	constrain function
h	specific enthalpy, kJ/kg
I	exergy destruction due to internal irreversibility, kJ
J	objective function
$\dot{m}$	mass flow rate, kg/s
p	pressure, Pa
P	power, W
$\dot{S}$	entropy rate, W/K
s	specific entropy, kJ/kg/K
$\dot{Q}$	heat transfer rate, W
$q_{f}$	specific cooling capacity of the gas, kJ/kg
$q_{\mathrm{iz}}$	heat penetration due to imperfect insulation, kJ/kg compressed gas
$q_{n}$	cooling loss due to the temperature difference at the hot end of the heat exchanger, kJ/kg
T	temperature, K
U	overall heat transfer coefficient, W/m^2^/K
W	work, kJ
w	specific mechanical work, kJ/kg
$w_{0}$	mechanical work consumption to obtain 1 kg of liquefied gas, kJ/kg liquid
y	fraction of liquefied gas, kg liquid/kg compressed gas
Subscripts	
0	environment, in equilibrium with the environment
C	Claude–Heylandt cycle
c	cold
cp	compressor
e	exit
ex	exergetic
f	forward gas stream
gen	generated
$G(O_{2}+N_{2})$	gaseous mixture of oxygen and nitrogen
h	hot
i	inlet, irreversibility, summation index.
iz	insulation
L	loss
L,1t	single-throttling Linde–Hampson cycle
L,2t	double-throttling Linde–Hampson cycle
$L\left(O_{2}\right)$	liquid oxygen
M	mean
min	minimum
n1	hot end of the first recuperative heat exchanger HX1
n2	hot end of the second recuperative heat exchanger HX2
n3	hot end of the throttling-stage heat exchanger HX3
opt	optimal
P	Pinch
r	returning gas stream
Rb	reboiler
t	throttle
t1	throttle valve upstream of the expander (TV1)
t2	throttle valve of the throttling stage (TV2)
Q	heat
$\Delta T_{n}$	temperature difference at the hot end of the heat exchanger
Superscript	
Q	associated with heat transfer
RC	Rectification Column
Greek Symbol	
Δ	difference
$\eta_{T,cp}$	compressor isothermal efficiency
η	efficiency
λ	lagrangian multiplier
Ψ	share of an exergetic loss or destruction in the fuel consumption

## Appendix A

Table A1 summarizes a quantitative comparison between the present exergetic model and published data for the Linde–Hampson and Claude (Heylandt) air-liquefaction cycles. The validation demonstrated that the trends and absolute magnitudes predicted by the present model were consistent with peer-reviewed references, confirming its physical reliability.

**Table A1:** Comparative performance indicators of cryogenic air-liquefaction cycles.

Indicator	Unit	Literature Linde–Hampson	Literature Claude–Heylandt	Present Work Claude–Heylandt
Liquefaction yield y	kg _liquid_/kg _air_	0.01–0.09 [7,37,38]	0.143–0.25 [37,38]	0.168–0.187
Specific energy consumption (SEC)	MJ/kg _liquid air_	4.98–22.67 [37,38]	1.06–5.9 [37,38]	3.58–3.96
Exergetic efficiency/Figure of Merit (FOM)	-	0.02–0.134 [7,37,38]	0.126–0.629 [37,38]	0.18–0.2
COP	-	0.029–0.077 [7,37,38]	0.128–0.3644 [37,38]	0.107–0.118
Performance Highlights	-	highest energy requirements, lowest liquid yield	lower energy requirements, higher liquid yield	lower energy requirements, higher liquid yield
